# Global Role of Crop Genomics in the Face of Climate Change

**DOI:** 10.3389/fpls.2020.00922

**Published:** 2020-07-16

**Authors:** Mohammad Pourkheirandish, Agnieszka A. Golicz, Prem L. Bhalla, Mohan B. Singh

**Affiliations:** Plant Molecular Biology and Biotechnology Laboratory, Faculty of Veterinary and Agricultural Sciences, University of Melbourne, Parkville, VIC, Australia

**Keywords:** domestication, genomics, climate change, crops, transcriptomics, abiotic stress

## Abstract

The development of climate change resilient crops is necessary if we are to meet the challenge of feeding the growing world’s population. We must be able to increase food production despite the projected decrease in arable land and unpredictable environmental conditions. This review summarizes the technological and conceptual advances that have the potential to transform plant breeding, help overcome the challenges of climate change, and initiate the next plant breeding revolution. Recent developments in genomics in combination with high-throughput and precision phenotyping facilitate the identification of genes controlling critical agronomic traits. The discovery of these genes can now be paired with genome editing techniques to rapidly develop climate change resilient crops, including plants with better biotic and abiotic stress tolerance and enhanced nutritional value. Utilizing the genetic potential of crop wild relatives (CWRs) enables the domestication of new species and the generation of synthetic polyploids. The high-quality crop plant genome assemblies and annotations provide new, exciting research targets, including long non-coding RNAs (lncRNAs) and cis-regulatory regions. Metagenomic studies give insights into plant-microbiome interactions and guide selection of optimal soils for plant cultivation. Together, all these advances will allow breeders to produce improved, resilient crops in relatively short timeframes meeting the demands of the growing population and changing climate.

## Introduction

The world will require a dramatic increase in food production in the next 30 years. Global food security is one of the key challenges of this century with the current human population of 7.7 billion expected to reach 8.6 billion in 2030 and 10 billion by 2050 (Tomlinson, 2013). The increase in population has led to an increase in urbanization, which is directly and indirectly, reducing our access to suitable land for agriculture (Satterthwaite et al., 2010). Simultaneously, the effects of climate change, including but not limited to increased temperature, changing patterns of rainfall, and increased levels of CO_2_ and ozone, impose further pressure on agriculture *via* drought and salinity that limit agricultural land and water use (Godfray et al., 2010).

Population growth is not the only reason we will need to increase food production. Significant income growth in rapidly developing economies gave rise to an emerging middle class, accelerating the dietary transition toward higher consumption of meat, eggs, and dairy products and boosting the need to grow more grain to feed more cattle, pigs, and poultry (Tilman and Clark, 2014). Agriculture in 2050 will need to produce almost 60–100% more food and feed than it is doing now (Tilman et al., 2011). This goal must be achieved despite the increase in global temperatures associated with climate change and growing scarcity of water and land, which are predicted to have significant impacts on the yield of all major crops.

In the last few centuries, plant breeders successfully used crossing and selection to improve the agronomic character of cultivated crops, such as wheat, maize, rice, barley, and others, resulting in dramatic increases in food production. However, agriculture has shifted to monoculture, resulting in the significant reduction of genetic diversity with today’s global agricultural food depending on a few key plant species (Khoury et al., 2014).

The genetic gains achieved by conventional crop breeding and advanced agronomic practices have led to more than a double increase in crop yields between 1960 and 2015. The development of dwarf varieties of rice and wheat coupled with greater use of synthetic fertilizers and irrigation led to the first green revolution. However, the yield increases due to the green revolution are declining and/or beginning to plateau for the major food crops (Grassini et al., 2013). After years of improvement, we are getting close to the final capacity of these few crops on yield and their tolerance to biotic and abiotic stresses. The current trend of annual yield increases for major crops of between 0.9 and 1.6% is insufficient to meet requirements in the near future (Ray et al., 2013). It has been estimated that about 2.4% annual yield gain is required to meet the global food demand (Ray et al., 2013). Thus, development of high-yielding climate change resilient crops with enhanced tolerance to water deficit, temperature, and biotic stresses is critical for increasing productivity to keep pace with the increasing human population.

The challenge of feeding the increasing human population under climate change conditions is unlikely to be met by conventional breeding technologies alone. Plant breeding must adopt new, multidisciplinary approaches to enhance the rate of genetic gain (Varshney et al., 2018). Fortunately, the science underpinning plant breeding is being revolutionized by the recent conceptual and technological innovations including the development of rapid, cheap sequencing technologies and the rise of genomics allowing for the detailed analysis of plant genomes and dissection of the genetic basis of agronomic traits. Genomics is now at the core of crop improvement, including the identification of genetic variation underlying differences in phenotypes, identification of additional sources of variation and novel traits, and characterization of molecular pathways involved in biotic and abiotic stress tolerance.

Recently the development of genome editing technologies, especially CRISPR/Cas9, opened new routes of fast and precise genome modification promising rapid translation of knowledge from the lab to the field. Genome editing allows introduction of insertions/deletions or an entirely new sequence at a desired location in the target genome (Scheben et al., 2017). Known genes controlling important traits can be selectively modified using genome editing, allowing for manipulation of phenotypes. In recent years, several genome edited crop plants entered final stages of commercialization in the United States of America including drought and salt tolerant soybean, *Camelina* with increased oil content, and waxy corn (Waltz, 2018).

Considering the urgent need for crop plant improvement and the new, exciting technological and conceptual developments, this review outlines the potential of genomic approaches (Table 1) for the development of climate change resilient crops.

**Table 1:** Summary of different approaches, which can be used to improve crop diversity and resilience.

Approach	Desired outcome
Using genomics to improve crop plant diversity and resilience	
Accessing genetic diversity of crop wild relatives (CWRs)	Diversification of the existing breeding resources
*De novo* crop domestication	Domestication of completely new crops using wild species
Engineering polyploidy	Controlled genome duplication or bridging the genomes of two related species
Harnessing plant-microbe interactions	Optimal choice of suitable crops for the specific soil type and geographic location
The challenge of climate change and plant diseases	Prediction of pathogen evolution and prevalence and deployment of suitable protective measures ahead of time
Genome editing for nutritionally enhanced crops	Editing of target genes to improve crop nutritional value
Accessing new breeding targets using genomic technologies	
Third-generation sequencing	Use of long sequencing reads for higher quality reference genome construction
Accurate gene prediction and functional annotation	Precise candidate gene identification
Analysis of the non-coding part of genome	Identification of new functional genomic sequences and breeding targets
Pangenome as a reference sequence	Inclusion of species-wide genomic variation in the analysis
Pairing genomics with other emerging technologies	
Machine learning and crop plant genomics	Use of artificial intelligence for crop genotype and phenotype prediction
Speed Breeding	Shortening the breeding cycle
High-throughput phenotyping	Increased resolution, accuracy and speed of plant phenotyping

## Using Genomics to Improve Crop Plant Diversity and Resilience

### Accessing Genetic Diversity of CWRs

**Figure 1:**
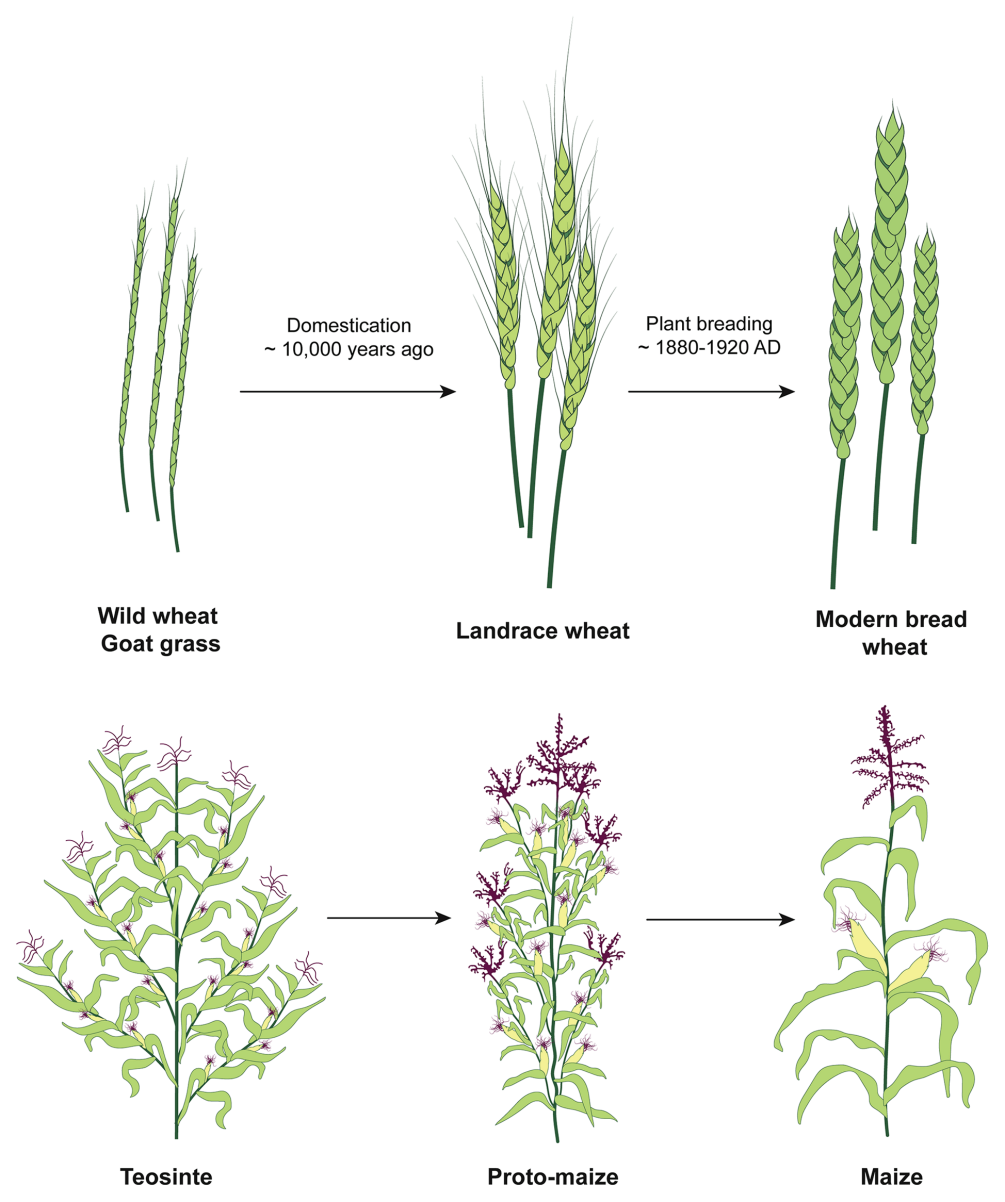
The schematic overview of the stages of wheat and maize domestication and improvement. In the course of domestication, ancient farmers developed landraces using wild populations. The improvement is an ongoing cyclical process by today’s breeders who identify desirable characteristics and develop strategies to combine the beneficial traits to obtain better varieties. The diversity of characteristics, such as biotic and abiotic stresses tolerance, is much higher in the wild compared to the modern varieties due to genetic bottlenecks associated with domestication.

Wild plants have survived under a changing climate for millions of years, during which they have been subjected to selective pressure by biotic and abiotic factors. This natural selection has led to the accumulation of genes allowing plants to resist, tolerate, or avoid extreme temperatures, draught, or flooding, as well as pests and diseases. However, during subsequent domestication, many of those, now important, traits and associated genetic material were lost, transforming some of the plants into our current remarkably productive crops with limited genetic diversity (Figure 1). The remainder of the genetic resources was left behind and mostly treated as a weed. Insights gained from the genome sequencing projects of different crops (Zhou et al., 2015b; Mascher et al., 2017; Appels et al., 2018; Springer et al., 2018; Zhao et al., 2018b) demonstrated the narrow germplasm of our modern crops and emphasized their vulnerabilities to climate change. However, all modern crop plants were domesticated from crop wild relatives (CWRs), which are still found in the wild, and provide a rich pool of genetic material, which is often excluded from the existing breeding programs (Brozynska et al., 2016).

Elite cultivated crops, such as wheat, maize, rice, and barley, are often dependant on farmer supplied resources, including water *via* irrigation, nutrition *via* fertilizers, and resistance to biotic stresses through the use of pesticides. This has led to the elite varieties becoming less resilient compared to their wild counterparts. In addition, strong artificial selection for a handful of crucial traits resulted in reduced diversity and restriction of the gene pool available within breeding programs. CWRs constitute an additional source of genetic diversity, which can be utilized during crop improvement programs, with as much as 30% of the increases in crop yields during the late 20th century being attributed to the use of CWRs in plant breeding programmes (^136,15^).

Over 1,500 CWRs of food crops have been identified as a potential source of genetic diversity for 173 globally important crops (Vincent et al., 2013). Advances in sequencing technologies facilitated construction of CRW reference genomes, which in turn can be used in comparative genomics analyses, allowing for the identification of novel genes controlling key traits (Brozynska et al., 2016).

**Figure 2:**
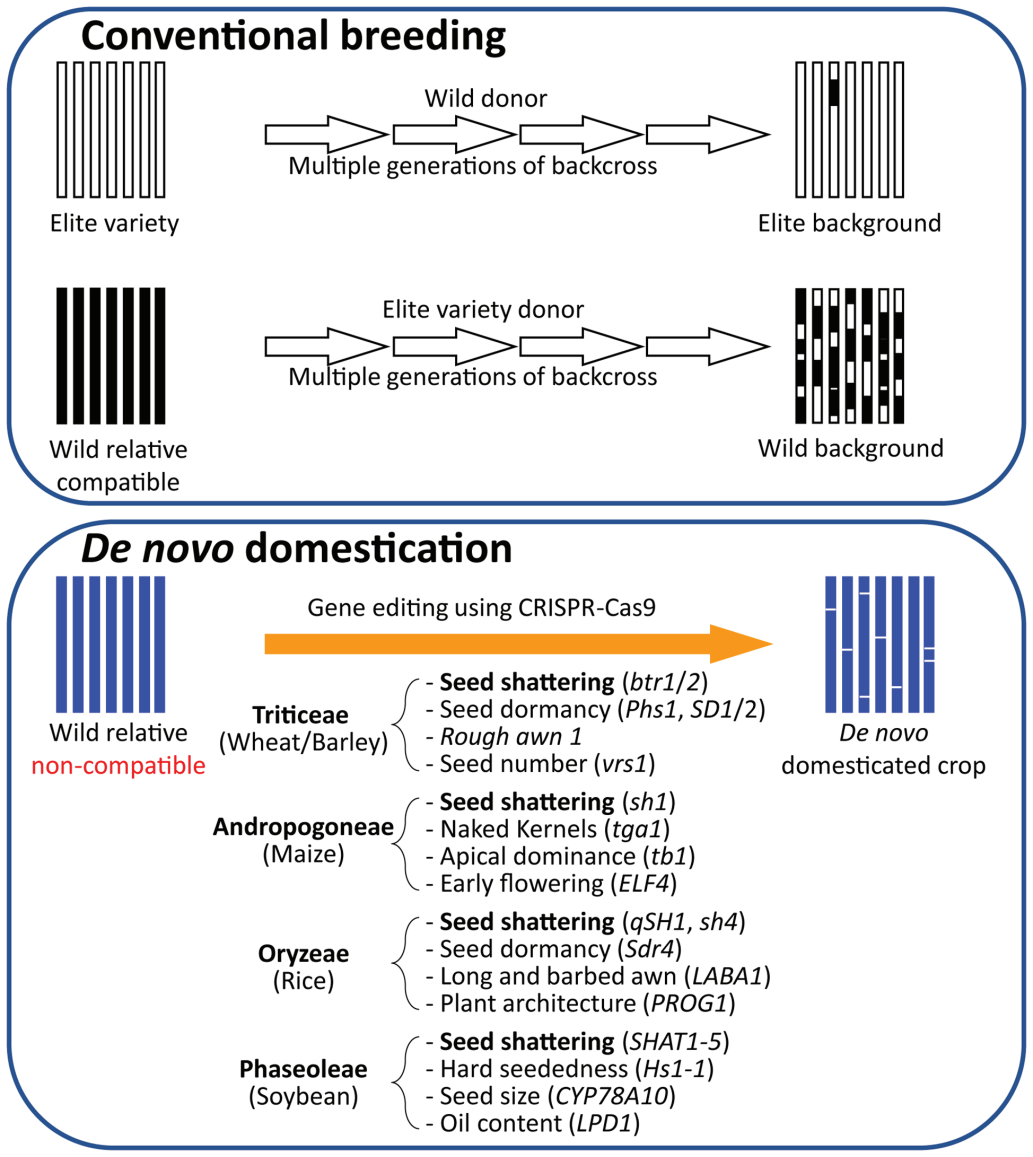
*De novo* domestication compared to conventional breeding. Comparison of the traditional breeding approach using backcrossing of the elite line with its wild relative with the molecular *de novo* domestication approach using the genome editing technique. Examples of potential target genes to be edited in the process of *de novo* domestication of wild relatives are indicated below the arrow.

Traditionally, the new genetic material was transferred from CWRs to crop plants by introgression of new genes into elite cultivar background (Figure 2; Dempewolf et al., 2017). Genomic resources have been widely used to speed up the process *via* marker assisted selection, including transfer of disease resistance genes in grape vine, apple, and banana (Migicovsky and Myles, 2017). Despite its obvious success, especially in transfer of major genes, the method is time consuming and restricted to sexually compatible species. For example, *Hordeum vulgare* (cultivated barley) has been extensively crossed with cross-compatible *Hordeum spontaneum* (wild progenitor), and there has been limited success crossing cultivated barley with *Hordeum bulbosum*, where chromosome segment from *H. bulbosum* can be transferred to the chromosomes of cultivated barley (Westerbergh et al., 2018). There are however 32 species in the genus *Hordeum*, including diploid, tetraploid, and hexaploid varieties (Bothmer et al., 1995), and the vast majority of *Hordeum* species cannot be used due to crossing barriers. However, once the candidate genes have been identified, transgenics and genome editing technologies can be used to transfer the desirable genetic material between species regardless of natural crossing barriers. To aid improvement, rich CWR genomic resources for many key crop species have been developed including soybean, rice, and maize.

*Glycine soja* – a wild relative of cultivated soybean (*Glycine max*) – has been shown to have a much more diverse gene pool compared to *G. max*, due to artificial selection during domestication and further loss as a result of modern breeding practices (Hyten et al., 2006; Kofsky et al., 2018). Wild and cultivated soybean differ in a number of agriculturally important traits, including pod shattering (Dong et al., 2014), determinate growth habit (Tian et al., 2010), and seed size (Zhou et al., 2015a; Kofsky et al., 2018). Additionally, it was shown that half of the annotated resistance-related sequences in *G. soja* were absent in both the landraces and cultivars (Zhou et al., 2015b). Despite the phenotypic differences, *G. soja* and *G. max* are cross-compatible, facilitating the transfer of desirable traits.

In maize (*Zea mays*), lowland teosinte (*Z. mays* ssp. *parviglumis*), highland teosinte (*Z. mays* ssp. *mexicana*), and the genus *Tripsacum* comprising nine species of warm-season, perennial grasses have been characterized as donors of important traits, which could be used for improvement (Mammadov et al., 2018). Genome-wide studies demonstrated that over 10% of the maize genome shows evidence of introgression from the *mexicana* genome, suggesting its contribution to adaptation and improvement (Hufford et al., 2013; Yang et al., 2017).

Rice (*Oryza sativa* L.) belongs to the genus *Oryza*, encompassing over 20 species, two of which are cultivated (*O. sativa* L. and *Oryza glaberrima* S.). The species are subdivided into several groups, and not all are cross-compatible. In recent analyses, *Oryza rufipogon*, a wild species believed to be the immediate progenitor of *O. sativa*, showed higher sequence diversity and harbored sequence and genes completely missing from the population of cultivated rice (Huang et al., 2012; Xu et al., 2012; Zhao et al., 2018b), highlighting the potential of its use for modern rice improvement.

In *Brassica*, a comparison of a CWR *Brassica macrocarpa*, with nine cultivated lines of *Brassica oleracea* showed that the former harbored unique disease resistance genes most likely lost during the domestication and improvement of elite *B. oleracea* germplasm (Golicz et al., 2016b).

The increasing abundance of genomic resources for CWRs will significantly aid future breeding efforts, helping identify the optimal crosses and genome editing targets.

### *De novo* Crop Domestication

Another strategy for utilization of the wild plant resources is new crop (*de novo*) domestication. The domestication syndrome refers to a unique collection of phenotypic traits associated with the genetic change of an organism from a wild progenitor to a domesticated one. Most of the changes linked to the domestication syndrome, such as grain dispersal in wheat, barley, and rice; apical dominance in maize; fruit size in tomato; and grain quality in wheat, result from modification of a single or few genes (Frary et al., 2000; Clark et al., 2004; Konishi et al., 2006; Uauy et al., 2006; Dubcovsky and Dvorak, 2007; Pourkheirandish et al., 2015, 2018). Also, most of them are due to a loss of function mutation in the causal gene (Komatsuda et al., 2007; Ramsay et al., 2011; Ishimaru et al., 2013; Pourkheirandish et al., 2015). For example, wheat, barley, rice, maize, and sorghum were selected for inflorescence that retained the grains, which made it easy to harvest. This characteristic results from the loss of function mutations in the genes controlling shattering (Konishi et al., 2006; Lin et al., 2012; Pourkheirandish et al., 2015). Similarly, a domestication associated NAC gene controlling pod shattering resistance has been identified in soybean (Dong et al., 2014). Advances in genomics provided the necessary platform to facilitate gene discovery and identification such as detection of genes associated with non-brittle rachis in pasta wheat and seed filling in maize using whole-genome sequencing (Sosso et al., 2015; Avni et al., 2017); smooth awn in barley using genotyping by sequencing (Milner et al., 2019); seed quality in soybean; and cutin responsible for water retention in barley using RNA sequencing (Li et al., 2012, 2017a; Gao et al., 2018).

The syntenic and orthologous gene relationships among plant genomes are well demonstrated (Devos, 2005; Tang et al., 2008). Synteny allows identification of homologous genes and has been used to identify genes with similar functions in related species (Pourkheirandish et al., 2007; Chen et al., 2009; Sakuma et al., 2010; Ning et al., 2013). For example, grain retention in both wheat and barley results from a mutation in homologous genes *brittle rachis* 1 (Pourkheirandish et al., 2018). The *brittle rachis* 1 homologues appear to have a similar role in grain dispersal in wild progenitors of wheat and barley. A loss of function mutation in this gene results in spike stiffness in the domesticated lines. As the same gene controls brittleness in both wheat and barley, *brittle rachis* 1 most likely evolved before the divergence of *Triticum* (wheat genus) and *Hordeum* (barley genus) over 5 Mya (Middleton et al., 2014). This suggests that the other non-domesticated species within *Hordeum* and *Triticum* that are not cross fertile with cultivated wheat and barley probably carry the *brittle rachis* 1, which controls their mode of grain dispersal. Recently a study involving crop plant species from multiple families used genome-wide association study (GWAS) to identify a domestication-related gene controlling seed dormancy in soybean and then showed that orthologs of this gene in rice and tomato also display evidence of selection during domestication. Analysis of transgenic plants confirmed the conservation of function in soybean, rice, and *Arabidopsis*, highlighting the power of comparative genomics in new domestication target gene identification (Wang et al., 2018a).

A pre-existing knowledge of target gene makes further crop domestication speedy and feasible. Domestication of a new crop species allows access to a novel gene pool with the potential for generating new crops, which are productive, resilient, and nutritious. Recent successes in wild tomato domestication by editing loci important for yield and productivity provide a proof of concept (Li et al., 2018; Zsögön et al., 2018). For example, targeting of *brittle rachis* 1 gene in any wild species of *Hordeum* or *Triticum* using gene editing would disrupt its function and result in a significant step toward domestication of a new species. It is important to note that the ease of genome editing and therefore its use for crop *de novo* domestication and other applications is related to plant ploidy. Gene knockout efficiency is lower in polyploids compared to diploids, as multiple alleles must be edited simultaneously to achieve a similar effect (Zhang et al., 2019b).

### Engineering Polyploidy

Polyploid plants possess three or more sets of homologous chromosomes stemming either from the duplication of a single genome (autopolyploidy) or hybridization followed by doubling of two diverged genomes (allopolyploidy; Comai, 2005). Many of the agriculturally important crop plants and staple food species are natural polyploids, including: bread wheat (allo-hexaploid; 6× = 42), pasta wheat (allo-tetraploid; 4× = 28), strawberry (allo-octaploid; 8× = 56), potato (auto-tetraploid; 4× = 48), and banana (auto-triploid; 3× = 33). Recent modeling work linked the occurrence of polyploidy to domestication (Salman-Minkov et al., 2016). Higher genome copy number masks deleterious mutations, increases the adaptive potential, and provides the opportunity for genes to gain new function. Thus, polyploidy is considered a major driver of evolution (Sattler et al., 2016). Induced polyploidy has also been used by breeders to develop new crops and flowers, such as triploid watermelon (seedless), hexaploid Triticale (a hybrid of wheat and rye), triploid tulips, roses, and many more ornamental flowers (Sattler et al., 2016). Polyploid plants tend to display hybrid vigor and improved abiotic stress tolerance (Chen, 2010; Tamayo-Ordóñez et al., 2016), with different manifestations of traits observed depending on the level of ploidy. For example, a study in *Arabidopsis*, which performed a rigorous comparison of plants with different somatic ploidy levels (2×, 4×, 6×, and 8×) observed significant differences in phenotypes (Corneillie et al., 2019).

The engineering of polyploid plants has been proposed as one of the routes for the generation of improved crop varieties (Katche et al., 2019). However, a better understanding of the causes and effects of polyploidy is a necessary prerequisite. Two major routes of polyploid plant formation are *via* unreduced gametes or somatic doubling (Ramsey and Schemske, 1998; Tamayo-Ordóñez et al., 2016). In laboratory conditions, polyploidy can be induced by application of antimicrotubule drugs such as colchicine. The viability of polyploid plants depends on stabilization of mitotic and meiotic divisions (Comai, 2005). Understanding of the molecular mechanisms behind cell cycle control, homologous chromosome pairing, and meiotic crossover formation is therefore paramount. Molecular mechanisms controlling cell cycle progression are deeply conserved and rely on cyclins (CYCs) and cyclin dependent kinases (CKDs). Previous studies in *Arabidopsis thaliana* identified seven classes of CDKs, named CDKA through CDKF, but CDKA and CDKB were identified as major drivers of cell cycle in plants (Menges et al., 2005; Tank and Thaker, 2011; Tamayo-Ordóñez et al., 2016). An extensive literature search compiled a list of over a 100 meiosis-related genes in *Arabidopsis* (Gaebelein et al., 2019). Comparative genomics approaches can be used to find orthologs of those genes in other species and perform further characterization. For example, a recent study of synthetic allohexaploid *Brassica* hybrids (2*n* = 6× = AABBCC) identified genomic regions associated with fertility, which harbored orthologs of *A. thaliana* genes involved in meiosis (Gaebelein et al., 2019).

In addition, plant genomes are known to undergo extensive structural rearrangements and methylation changes upon polyploidization. A study of resynthesized *Brassica napus* lines demonstrated extensive restructuring of the merged genomes in the early generations following hybridization (Szadkowski et al., 2010). Many hybrids and recent allopolyploids display genome dominance, resulting in sub-genome biases in gene content and expression (Bird et al., 2018). Genomics can be used to track post-hybridization structural re-arrangements and the establishment of sub-genome dominance to better understand plant genome evolution post-hybridization (Edger et al., 2017). It can also help predict the optimal combination of different wild species to construct new synthetic crops that can diversify our agriculture and bring resilience to climate change.

**Figure 3:**
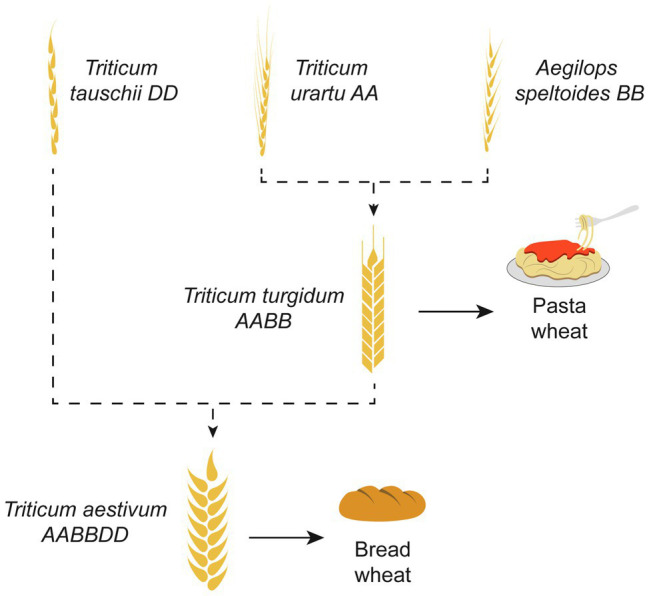
The origin of bread wheat. Bread wheat (*Triticum aestivum*), is an allo-hexaploid originated *via* multiple hybridizations. The most accepted hypothesis of its origin is based on the hybridization of *Triticum urartu* (AA; 2× = 14) and *Aegilops speltoides* (BB; 2× = 14), resulting in tetraploid pasta wheat (AABB; 4× = 28). At the next step, hybridization of the tetraploid wheat with *Aegilops tauschii* (DD; 2× = 14) resulted in the emergence of the hexaploid bread wheat (AABBDD; 6× = 42).

**Figure 4:**
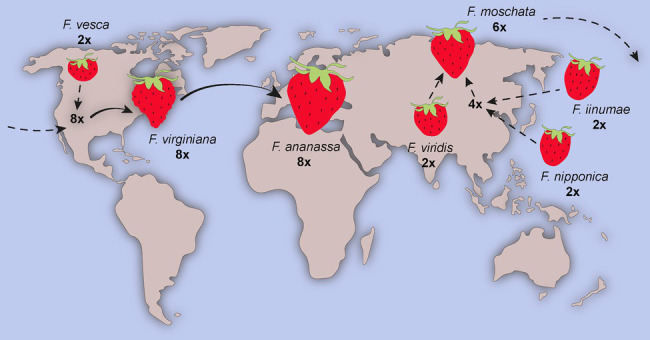
The origin of strawberry. Strawberry is an allo-octoploid originated from multiple hybridization events in Eurasia and America. Modern variety *Fragaria × ananassa* resulted from cross between two octoploid genotypes *Fragaria virginiana* and *Fragaria chiloensis*. Figure adapted from Bertioli (2019).

As an example, bread wheat (*Triticum aestivum*), a major crop accounting for 20% of world daily food consumption, is an allohexaploid plant originated *via* multiple hybridizations. The most accepted hypothesis of its origin is demonstrated in Figure 3 (Haider, 2013). Because the bread wheat carries genes of three different genomes (A, B, and D), it is robust and has been able to adapt to different climatic zones. Today, bread wheat (AABBDD), which originated from fertile crescent (30–35°N), can grow from Sweden (65°N) to Argentina or New Zealand (45°S), a cultivation zone much broader than that pasta wheat (AABB; Feuillet et al., 2008). Another example of a widely known polyploid plant is the octoploid strawberry (Edger et al., 2019). The modern strawberry arose from a series of hybridization events between diploid, tetraploid, and hexaploid species spanning Eurasia and North America (Figure 4).

### Harnessing Plant-Microbe Interactions to Boost Agricultural Output

**Figure 5:**
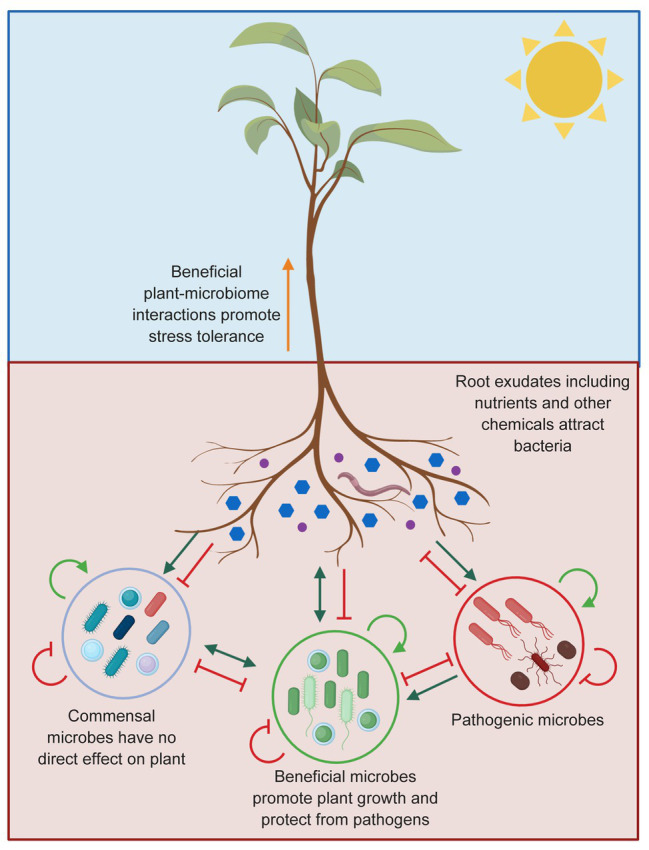
Plant-microbe interactions in the rhizosphere. Plants can influence the composition of microbiome surrounding plant roots through exudation of compounds that stimulate (green arrows) or inhibit (red blocked arrows) microbes. A wide range of pathogens living in the soil can also affect plant health. Being able to attract the beneficial microbes will limit the success of the pathogenic microbes due to resource competition or by enhancing the plant immune system. The commensal microbes do not affect the plant or the pathogen directly. Microbiome-plant interactions are presented as described by Berendsen et al. (2012).

Microbes which live within (endosphere) and surrounding plant roots in the soil (rhizosphere) have a significant impact on the host, including health and fitness, productivity, and responses to climate change (Wei et al., 2019). Plants and microbes interact *via* signaling molecules originating from both organisms (Leach et al., 2017; Chagas et al., 2018). Some bacterial communities have been shown to manipulate the plant potential to use soil resources, promote plant biotic and abiotic stress tolerance, and stimulate growth and nutrient uptake (Mendes et al., 2011; Berendsen et al., 2012; Fellbaum et al., 2012; Fitzpatrick et al., 2018; Paredes et al., 2018). At the other end of the spectrum, pathogenic microbes also exist, which negatively affect plant health (Figure 5; Pusztahelyi et al., 2015; Chagas et al., 2018).

Soil microorganisms are in constant competition to accumulate around the root and access the plant secreted carbohydrates (Venturi and Keel, 2016). Plants and microbes evolved together, resulting in beneficial microbes being attracted to a specific root exudate profile and forming a community of microbes in the rhizosphere (Garbeva et al., 2008). Plant species can therefore shape the composition of their rhizosphere’s microbiome. Crops grown in the soils with microbial profiles similar to their native environment are expected to have a better chance of forming beneficial plant-microbiome interactions (Pérez-Jaramillo et al., 2018), promoting tolerance to biotic and abiotic stress. The advances in genomic technologies resulted in the sequencing of numerous soil microorganisms and improvement of our understanding of the soil microbial communities (Jansson and Hofmockel, 2018). For example, the availability of genomic sequences for nitrogen-fixing and phosphate-solubilizing bacteria expanded significantly (Zekic et al., 2017; Basenko et al., 2018; Jeong et al., 2018; Ormeño-Orrillo et al., 2018). Combined analysis of microbiome genomic and metabolomic data provides an accurate tool necessary to understand plant-microbe interactions and predict the most favorable crop plant–soil microbiome combinations, allowing for mapping of suitable crops to specific locations.

### The Challenge of Climate Change and Plant Diseases

Crop plant pathogens are considered a major threat to modern agriculture (Oerke and Dehne, 2004; Savary et al., 2012; Nelson et al., 2018). The ongoing battle between plants and pathogens resulted in their co-evolution and shaped the genetic diversity of both (Jones and Dangl, 2006; Tiffin and Moeller, 2006; Dodds and Rathjen, 2010; Hartmann et al., 2017). Diseases generally result from a specific interaction between host and pathogen (Veresoglou and Rillig, 2014; Põlme et al., 2018). For example, the wheat leaf rust pathogen *Puccinia triticina*, one of the most common diseases of wheat globally, does not affect rice, maize, or any other crop. World-wide over-cultivation of a few crops (wheat, maize, rice, soybean, and barley) with low genetic diversity has led to the increased pathogen inoculum and accelerated pathogen evolution, promoting its spread globally (Savary et al., 2019).

Climate change affects the epidemiology of pathogens at specific locations and the geographic distribution of plant diseases (Barford, 2013; Chakraborty, 2013). Increasing the crop plant diversity by cultivation of orphan crops and the domestication of new crops will result in reduced selective pressure on pathogen populations; thus, the life of genetic resistance is expected to be longer (Cook, 2006; Hajjar et al., 2008; Storkey et al., 2019). The life extension of genetic resistance could be an effective and ecologically sustainable way to control diseases. Climate change affects not only the crop but also the pathogen survival and reproduction. One of the expected impacts of climate change on plant disease is the migration of pathogens to latitudes beyond their historical range, examples of which have already been documented (Barford, 2013; Chakraborty, 2013). An increase in temperature would result in pathogen movement and spread of disease further from north in the northern hemisphere and south in the southern hemisphere to geographical locations in which they previously have not been able to reproduce effectively nor infect the plant. Recent genomic advances have resulted in the prediction and isolation of several resistance genes from crops and the identification of the corresponding genes from the pathogen (Fu et al., 2009; Mago et al., 2015; Moore et al., 2015; Sperschneider et al., 2015; Krattinger et al., 2016; Wan et al., 2019). These advances have provided a snapshot of resistance mechanisms that crops have developed during the long co-evolutionary history. The discovery of the genes underlying resistance has led to an improved understanding of their molecular function and established an entry point for studies of the defense pathways.

In addition, genome sequencing provides a rapid method of pathogen identification (Boykin et al., 2019), outbreak progression, and tracking of its spread to new locations. In fact, the development of third-generation sequencing technologies, especially Oxford Nanopore, resulted in the introduction of small, affordable, mobile sequencing instruments perfectly suited for in-field diagnostic system. Oxford Nanopore MinION technology has already been used for real-time diagnostics of human pathogens including Ebola (Quick et al., 2016) and Zika viruses (Faria et al., 2016) with protocols for identification of plant pathogens and pests under active development. For example, a recent proof-of-concept study has shown that using portable sequencing technology diagnostic test, it is possible to deliver test results within 48 h and, thus, greatly reduce the risk of community crop failure (Boykin et al., 2019).

### Genome Editing for Nutritionally Enhanced Crop Production

Augmentation of crop nutritional value plays a central role in ensuring global food security. Breeding of crops for enhanced nutrient content has been a long standing goal of plant research (DellaPenna, 1999; Welch and Graham, 2002). Plants are a key source of macro‐ and micro-nutrients, but many of the staple foods, including cassava, wheat, rice, and maize are poor source of some macro-nutrients and many essential micro-nutrients (DellaPenna, 1999). However, nutrient profile can be altered by manipulation of biochemical pathways involved in macro‐ and micro-nutrient biosynthesis. Advances in genome sequencing and annotation provided the necessary resource to identify the candidate genes involved in plant metabolism. As a result, genome editing technologies could be used to modify nutritional profiles of crops, for example producing soybeans with high oleic acid and low linoleic acid content (Haun et al., 2014; Demorest et al., 2016) and reducing anti-nutritional phytic acid content in maize (Liang et al., 2014). Nutritional enhancement of crops can also be achieved using transgenic technologies (Hefferon, 2015). In addition, genome editing facilitated *de novo* domestication of new nutrient rich crops could lead to a more diversified and healthier diet.

## Accessing New Breeding Targets Using Genomic Technologies

### Third-Generation Sequencing for Improved Reference Genomes

The beginning of the twenty-first century saw rapid development of new sequencing methods. Second-generation sequencing technologies, including Illumina, allowed assembly of over 200 plant genomes (Chen et al., 2018) with much more ambitious plans of generating 10,000 draft genome assemblies by 2025 (Cheng et al., 2018). The main challenge posed by second-generation sequencing technologies was short-read length, making them unable to bridge over long stretches of repetitive sequences, resulting in fragmented assemblies. However, the introduction of third-generation sequencing and long reads produced by PacBio and Oxford Nanopore now allows for chromosomal level assemblies of plant genomes (Belser et al., 2018). The long-read sequencing technologies are often combined with optical mapping and conformation capture, achieving draft genomes of unprecedented contiguity (Belser et al., 2018; Shi et al., 2019). Importantly, the sequencing strategy used and the resulting contiguity and completeness of the assembly have been shown to impact downstream evolutionary and functional analyses. For example, comparative analysis of two *Brassica rapa* assemblies, one built using Illumina sequencing data and the other one using a PacBio, optical mapping (BioNano) and conformation capture (Hi-C) revealed that the latter harbored ~3,000 assembly specific genes as well as over 500 previously unidentified transposable element (TE) families (Zhang et al., 2018). The availability of high-quality, chromosome scale genome assemblies substantially improves the accuracy of the downstream genomic analysis, including gene and regulatory region annotation, GWAS, gene expression quantification, and homologue detection.

### Accurate Gene Prediction and Functional Annotation for Precise Candidate Gene Identification

The explosion of plant genome sequencing was accompanied by extensive annotation efforts aiming to generate a comprehensive catalog of gene models for a given species. Gene model is defined as a region of the genome, which is believed to be transcribed into protein-coding messenger RNA (mRNA) or one of the classes of non-coding RNAs (ncRNA; Schnable, 2019). Gene models are often built using a combination of *ab initio* gene prediction and homology-based methods that take advantage of sequence similarity to known transcripts or proteins (Campbell et al., 2014; Klasberg et al., 2016). Early on gene expression evidence was mostly derived from expressed sequence tags (ESTs) and extended by full-length sequencing *via* cloning followed by Sanger sequencing. Later, the information was supplemented by RNASeq data from diverse tissues, and it was shown that gene models and isoforms with highly tissue-specific expression were underrepresented in exiting annotations (Cheng et al., 2017; Golicz et al., 2018b; Van Bel et al., 2019). Currently, addition of long reads generated by PacBio or Oxford Nanopore sequencing technologies allows for recovery of full-length transcripts, providing new insights into the extent of alternative splicing and transcriptome diversity (Cook et al., 2019). Annotation of loci harboring non-coding transcripts is also becoming routine, further improving our understanding of the complexity of plant transcriptomes (Van Bel et al., 2019).

Despite the availability of genome annotations, functional characterization of annotated genes, which allows for the direct connection between genome and phenome, poses a key challenge in molecular breeding pipelines (Scheben and Edwards, 2018). In the key experimental model plant species, *A. thaliana*, >90% of genes have been annotated with putative functions and ~50% of genes have annotation supported by experimental evidence (Van Bel et al., 2019). However, for most of the crop plants, gene functional annotations rely on homology-based inference and are performed by transfer of annotation from most similar genes in model plants like *Arabidopsis* and rice, with very little direct experimental support. Annotation transfer is further complicated by plant evolutionary history, where successive rounds of polyploidy and subsequent diploidization lead to gene redundancy, differential loss, and neo‐ and sub-functionalization (Jiao and Paterson, 2014; Salman-Minkov et al., 2016). However, rapid progress in application of CRISPR/Cas9 genome editing will soon allow construction of genome-wide mutant libraries for key crops, significantly contributing to the functional annotation efforts. In fact, such libraries are already available for rice (Lu et al., 2017; Meng et al., 2017). Integrative genomics approaches have also been used to facilitate discovery of top candidates. For example, specialized databases integrating genotypic, phenotypic, and association data have been developed for rice (SNP-Seek), soybean (SoyBase), and wheat (T3; Grant et al., 2010; Blake et al., 2016; Mansueto et al., 2017). Beyond specialized database, tools like KnetMiner and MCRiceRepGP were developed aiming to rank candidate genes involved in biological processes of interest using multicriteria decision analysis (Hassani-Pak and Rawlings, 2017; Golicz et al., 2018b).

### Non-coding Part of Genome as a Reservoir of New Breeding Targets

Only several percent of most large crop plant genomes encode protein-coding genes and the remainder is made up of non-coding sequences. For a long time, the non-coding stretches of DNA were considered to have little function; however, recent technological and conceptual developments revealed that plant genomes encode thousands of potentially functional ncRNAs as well as prevalence of distant regulatory elements including enhancers (Weber et al., 2016). The ncRNAs encompass several classes of transcripts, including not only the relatively well characterized ribosomal RNAs (rRNAs), transfer RNAs (tRNAs), small nucleolar RNAs (snoRNAs), and micro RNA (miRNAs) but also much more poorly understood long non-coding RNAs (lncRNAs). LncRNAs are transcripts over 200 base pairs in length without discernible protein coding protentional, identified from RNASeq data, and have been shown to be involved in a range of biological processes, including flowering time regulation, stress tolerance, and gamete formation (Golicz et al., 2018a). At least some of the lncRNAs are likely to be functional, as evidenced by mutant phenotypes of knock-outs of newly discovered lncRNAs (Huang et al., 2018). Interestingly, lncRNAs have a strong bias toward transcription in reproductive tissues (Zhang et al., 2014; Golicz et al., 2018b; Johnson et al., 2018), suggesting involvement in plant sexual reproduction, a critical process affecting flowering, fruit, and grain formation. Newly characterized lncRNA, which affect important traits, can become genome editing targets. For example, a rice lncRNA LDMAR was shown to be involved in control of photoperiod-sensitive male sterility (PSMS), a key trait which contributed to the development of hybrid rice (Ding et al., 2012).

Another promising category of non-coding DNA sequences are cis-regulatory elements (CREs, promoters and enhancers/silencers), capable of recruiting transcription factors and promoting gene expression. Changes in CREs are considered one of the key evolutionary mechanisms underlying, for example, emergence of novel morphological forms (Stern and Orgogozo, 2008; Weber et al., 2016) and the divergence of cis-regulatory regions associated with domestication underscore their important roles in control of traits targeted by artificial selection (Lemmon et al., 2014; Wang et al., 2017). Several enhancers have been identified that modulate the expression of genes involved in the control of important traits, like anthocyanin content in maize and flowering time in *Arabidopsis* (Chua et al., 2003; Louwers et al., 2009; Adrian et al., 2010). SNPs corresponding to the different rapeseed ecotype groups were also identified in the promoter regions of FLOWERING LOCUS T and FLOWERING LOCUS C orthologs (two key genes controlling flowering time; Wu et al., 2019). An SNP corresponding to spatial expression of a homeobox transcription factor was selected for during the selection of non-shattering rice (Konishi et al., 2006). An insertion of TE in the CRE of *teosinte branched* 1 gene was discovered as the reason for apical dominance in maize also selected in the course of plant domestication (Studer et al., 2011). In the last few years, a significant progress has been made in identification of plant CREs with studies of the model plant species *Arabidopsis* as well as rice, maize, and cotton (Zhang et al., 2012; Pajoro et al., 2014; Zhu et al., 2015; Rodgers-Melnick et al., 2016; Oka et al., 2017; Wang et al., 2017; Bajic et al., 2018; Tannenbaum et al., 2018; Zhao et al., 2018a; Yan et al., 2019). The rapid developments are due to adoption of DNase-Seq and ATAC-Seq techniques in plant research, which measure DNA “openness” as a proxy for the accessibility of DNA to transcription factors, RNA polymerase, and other protein complexes involved in gene expression (Pajoro et al., 2014; Wang et al., 2016). Improved understanding of the function of the non-coding elements of the genome will provide a new, yet untapped pool of breeding targets.

### Beyond Single Reference Genomics – The Pan-Genome Approach

**Figure 6:**
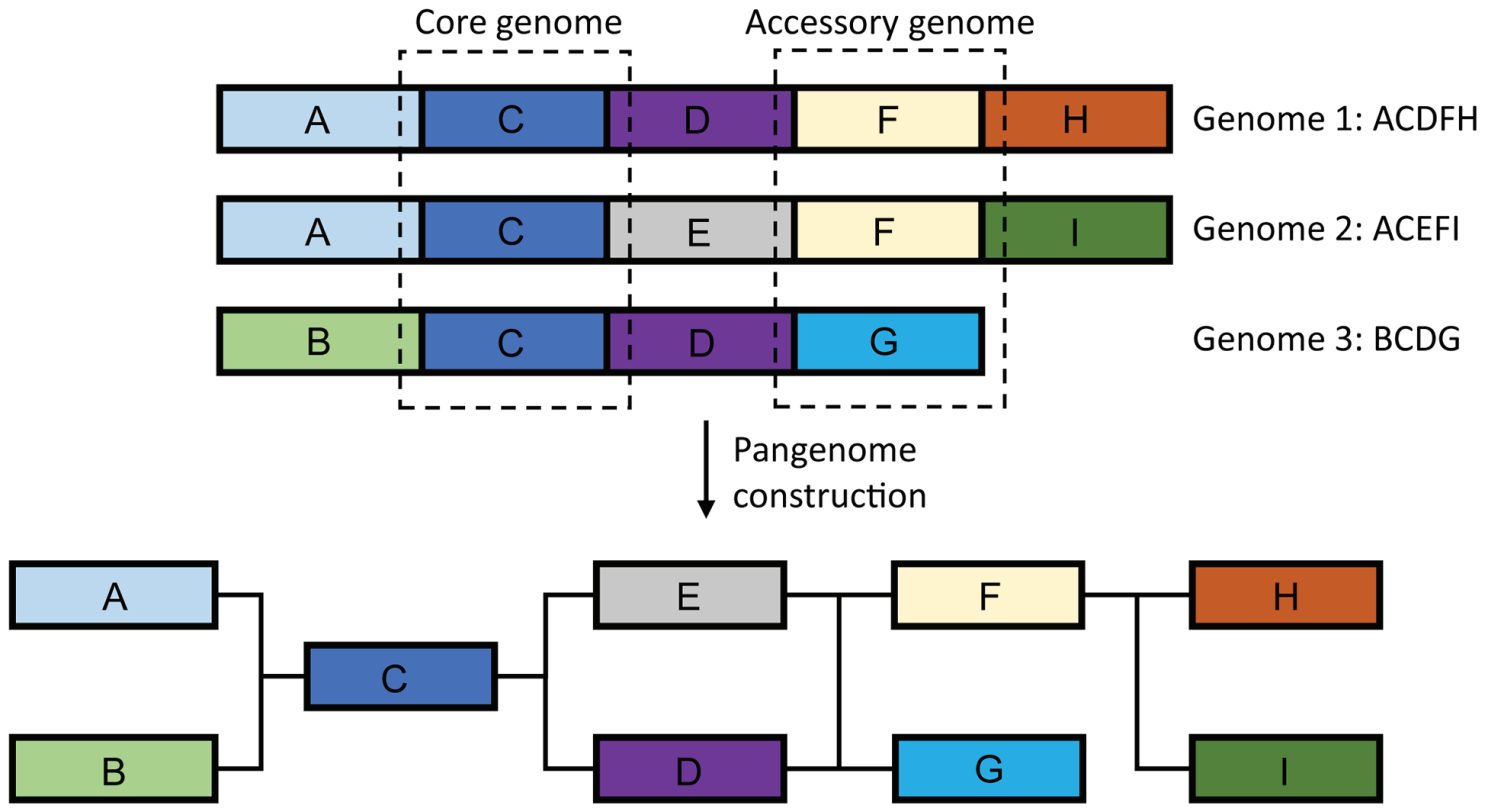
Schematic representation of the plant pangenome. Pangenome represents the entire genomic sequence found in the species.

Generation of the reference genomes and subsequent large-scale re-sequencing of hundreds to thousands of individuals per species revealed extensive genomic diversity, including large-scale presence/absence variation (Golicz et al., 2016a; Varshney et al., 2017, 2019; Fuentes et al., 2019; Wu et al., 2019). As our knowledge of genomic variation increased, it become apparent that a single reference sequence is insufficient to represent the extent of genomic variation found within species, resulting in the introduction and adoption of the pangenome concept (Figure 6; Golicz et al., 2020). Pangenome represents the entirety of the genomic sequence and gene content found within a species rather than a single individual. First introduced in bacteria (Tettelin et al., 2005), it is highly relevant to plant research with more than 50% of genes in some species being variable (accessory), found in some individuals but not others (Golicz et al., 2020). Pangenomes have been constructed for key crop species, such as rice, soybean, bread wheat, and oilseed rape (Li et al., 2014; Golicz et al., 2016b; Contreras-Moreira et al., 2017; Gordon et al., 2017; Montenegro et al., 2017; Zhou et al., 2017; Hurgobin et al., 2018; Ou et al., 2018; Zhao et al., 2018b; Gao et al., 2019; Zhang et al., 2019a). Plant accessory genes have been shown to be over-represented in functions related to signaling and disease resistance as well as abiotic stress response (Golicz et al., 2016b; Montenegro et al., 2017; Hurgobin et al., 2018; Wang et al., 2018b), perhaps contributing to environmental adaptation and phenotypic plasticity and providing promising targets for crop improvement. Especially, since some of the accessory genes may be completely missing from the elite germplasm.

In addition, the pangenome offers a natural replacement for the current paradigm of using a single reference genome, as the choice of the reference affects downstream genomic analyses, including GWAS and gene expression quantification (Gage et al., 2019). Using pangenome as a reference improves read mapping and variant calling accuracy (Eggertsson et al., 2017; Garrison et al., 2018; Kim et al., 2019; Tian et al., 2019). The adoption of the pangenome reference will also allow the inclusion of variants beyond SNPs in GWAS. Several studies in both plants and humans showed that the inclusion of structural variants in association studies could help identify causal variants (Chiang et al., 2017; Fuentes et al., 2019). For example, the use of sequence presence/absence variation allowed the identification of missing quantitative trait locus (QTLs) associated with disease resistance in oilseed rape (Gabur et al., 2018).

## Pairing Genomics with Other Emerging Technologies to Maximize Their Potential

### Machine Learning and Crop Plant Genomics

Almost all aspects of genomic analyses can now be supported by the development and implementation of machine learning algorithms. Machine learning algorithms find new patterns and “learn” the necessary predictive features from the data, rather than rely on pre-existing criteria. This property makes them suitable for analysis of complex, multilayer datasets, where expert knowledge is incomplete or inaccurate, and when the amount of data is too large to be handled manually (Yip et al., 2013). Several promising applications of machine learning to plant genomics exits. As discussed above, the functional non-coding portions of plant genomes remain largely poorly understood. In animal research, machine learning and deep learning based methods have been particularity successful in genomic feature annotation, including regulatory regions like promoters, enhancers, and transcription factor binding sites (Xu and Jackson, 2019). The use of machine learning improved the quality of feature annotation, helped uncover the underlying sequence characteristics of the regulatory regions, and even allowed prediction of variant impact (Zhou and Troyanskaya, 2015; Kelley et al., 2016). However, the limited availability of large-scale epigenetic modification and chromatin accessibility datasets may delay similar studies in crop plants. While the lack of suitable datasets may be hampering regulatory region annotation, hundreds of sequenced and assembled genomes are readily available for comparative analyses. Identification of conserved and unique elements is one of the primary aims of comparative genomics. To date, sequence comparisons are mostly based on local or whole-genome alignments and limited by sensitivity of alignment tools. However, machine learning algorithms are being developed, which are capable of computing probability of sequence conservation for any query of interest (Joly-Lopez et al., 2016; Li et al., 2017b), providing new, exciting avenues for plant comparative genomics. Finally, plant phenotyping has legged significantly behind genotyping, requiring considerable resources and specialized equipment (Scheben and Edwards, 2018). One proposed application of machine learning is the prediction of phenotype from genotype and complementation of the more traditional genomic prediction models (Ma et al., 2018). Taken together, machine learning methods have the potential to add significant value to the existing genomic resources and methodologies.

### Speed Breeding to Accelerate the Development of New Crops

Advances in molecular and genomic technologies resulted in isolation and characterization of many agronomically important genes, for example ones controlling seed shattering, dormancy, increasing seed number, and size (Doebley et al., 2006). Improved understanding of the molecular function of these genes makes the new crop domestication and improvement of orphan crops feasible. However, the generation of new crops or improved crop varieties using traditional breeding techniques requires a lengthy process of recurrent selection, which can take many years (Gorjanc et al., 2018). One of the limiting factors in the process is the plant generation time, from seed germination to the harvest. The plant generation cycle takes up to 4 months in wheat and barley and even longer in others (Watson et al., 2018). Domestication of new crops would require numerous generations to stack the edited genes before crop release. Speed breeding is a procedure, which accelerates crop generation time by changing growth conditions, such as day length and temperature (Hickey et al., 2017). Growing long-day species under extended photoperiod (22 h light/2 h dark) and controlled temperature stimulates rapid flowering and maturation. The technology successfully shortened the plant generation time of some of the world’s major agri-food crops, such as bread wheat, pasta wheat, barley, and canola (Watson et al., 2018). Production of up to six generations for wheat and barley is documented using speed breeding, which is much more efficient compared to two generations per year in traditional methods. Speed breeding protocols have also been successfully applied to orphan crops, such as chickpea, peanut, grass pea, lentil, and quinoa (O’Connor et al., 2013; Chiurugwi et al., 2019). The successful application of speed breeding to orphan crops indicates its flexibility and possible application for new crop domestication. A combination of speed breeding with our current knowledge about the target genes and genomic tools such as precision genome editing by CRISPR would make the new crop domestication feasible in a short time. Speed breeding can also be paired with genomic selection (GS), allowing further reductions in plant breeding cycles. GS is a modern breeding technology, which uses genome-wide markers to estimate the breeding values (EBV) and allows simultaneous selection for multiple traits. A recent study combined multivariate GS and speed breeding for yield prediction in spring wheat (Watson et al., 2019). Even though the current speed breeding protocols are limited to the long-day species, new protocols are expected for the short-day crops in the near future. Coupling speed breeding with genomics will make the GS for breeding and *de novo* domestication feasible.

### High-Throughput Phenotyping

Plant phenotyping refers to the measurement of any morphological or physiological characteristics of plants. The phenotype can result from the action of individual genes, gene-by-gene, or gene-by-environment interactions. Many agronomically essential traits, such as yield and its components and drought/salt tolerance, are controlled by multiple genes with small effects and their interactions with the environment (Mickelbart et al., 2015). For practical reasons, many research groups focus on a controlled environment to grow plants and study their response to biotic and abiotic stresses (Velásquez et al., 2018). This includes stress induced by temperature, humidity, light, and other environmental factors. However, in farming, the environment and microclimate change dynamically during the day and affect the plant unevenly, for example due to shading. Moreover, controlled light conditions are hardly equivalent to the irradiance levels and spectral quality typical of natural sun conditions. There is a great need to study plant stresses in dynamic environmental conditions to thoroughly understand the complete picture of plant-stress responses. As the genotypic information is now available for hundreds or thousands of breeding lines in different species, collection and analysis of the corresponding high-throughput phenotyping data is one of the significant tasks ahead (Araus et al., 2018).

High-throughput phenotyping platforms, which employ robotics and spectral-based imaging technologies, are rapid and reliable (Galvez et al., 2019). The main limitation is the controlled environment, which is different from the natural growth conditions in the field. The introduction of hyperspectral imaging technology combined with drones and manned aircrafts provides an opportunity for high-throughput in-field phenotyping of traits, such as canopy temperature, chlorophyll fluorescence, as well as other biochemical plant characteristics (Camino et al., 2019). This technology increases the resolution and accuracy of measurements and is becoming cost-effective. The main challenge of using airborne platforms would be the analysis of large quantity of data in a short time frame (Singh et al., 2016; Taghavi Namin et al., 2018). However, machine learning based methods have shown promise in high-throughput phenotyping data processing. In-field high-throughput phenotyping is perfectly suited for evaluation of the complex physiological traits such as abiotic stresses tolerance.

## Conclusion

Recent advances in genome sequencing, assembly, and annotation allowed unprecedented access to crop plant genomic information. High-throughput phenotyping techniques have been significantly advanced through the introduction of hyperspectral cameras and specialized processing software. Integration of genomic and phenomic data provides an opportunity to identify new agronomically relevant genes and characterize their functions. This knowledge has direct practical implications and can be translated to crop plant improvement using genome editing. While genome editing is currently applied in major crops and model plants, the technique has the potential to accelerate *de novo* domestication and allow rapid improvement of orphan crop plants, targeting the current and future climate challenges. The success of genomics in crop improvement is also influenced by the type of trait under investigation. For example, traits strongly affected by the environment and the interaction between genotype and the environment are more challenging to study and modify.

**Figure 7:**
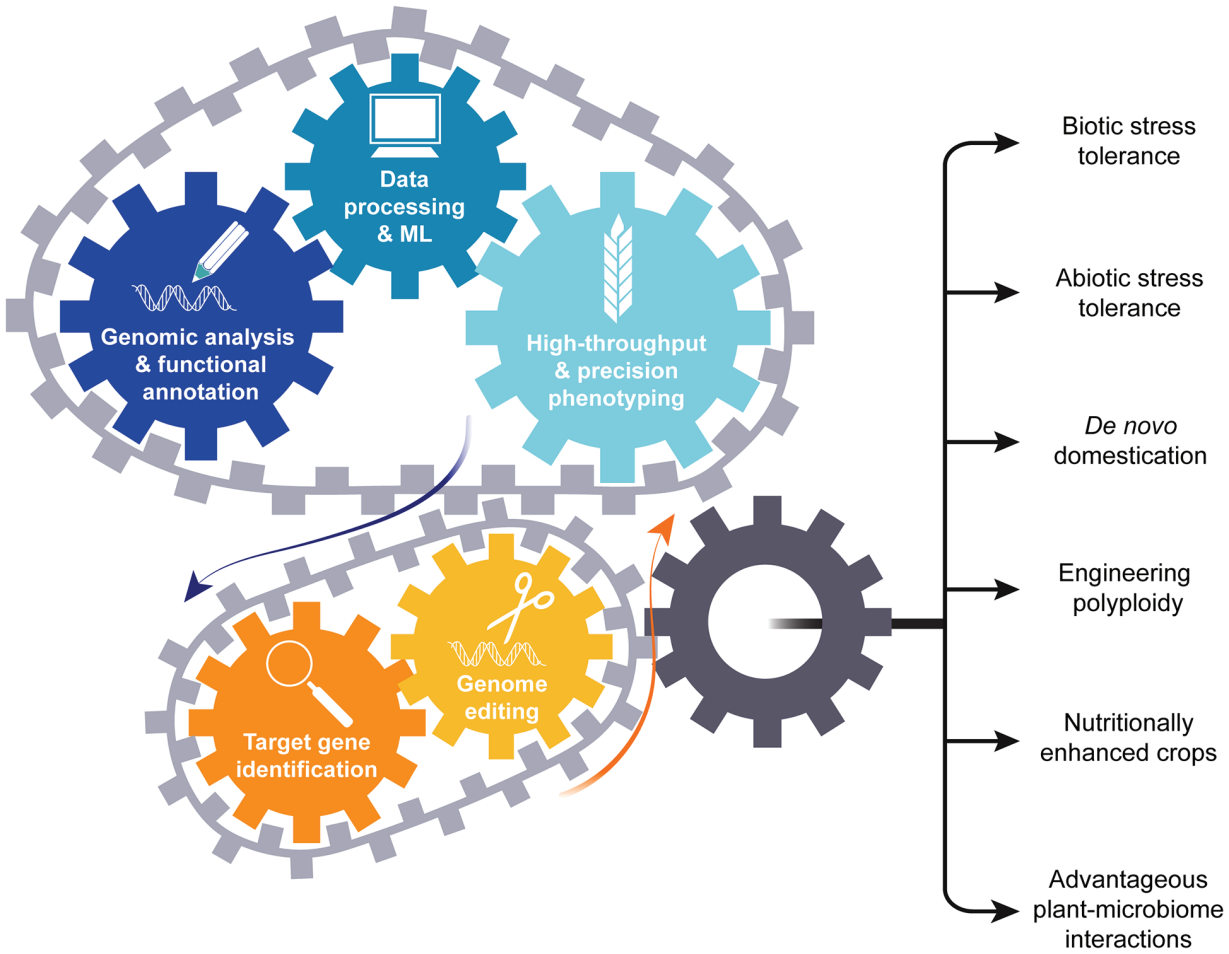
Genomics drives development of climate change resilient crops. Knowledge obtained from genomics and high-throughput phenotyping drives candidate gene selection. Candidate genes can then be modified using genome editing resulting in generation of improved crop types.

Disease resistance and dwarfing genes were introduced into crops such as wheat and rice during the green revolution (Khush, 2001). Breeders developed the high yielding varieties using the extra supply of nitrogen fertilizers in the presence of sufficient water under the climate conditions of the 1950–1960’s. The equation is different today as climate change causes water shortages and temperature increases. However, the information gained from genomics and phenomics will drive candidate gene identification and enable genome editing (Figure 7), initiating the new crop plant breeding revolution.

## Author Contributions

MP and AG contributed equally. All authors contributed to the article and approved the submitted version.

## Conflict of Interest

The authors declare that the research was conducted in the absence of any commercial or financial relationships that could be construed as a potential conflict of interest.
